# Patients' Perspectives and Attitudes About the Relationship Between Fixed Orthodontic Treatment and Oral Hygiene

**DOI:** 10.7759/cureus.68178

**Published:** 2024-08-30

**Authors:** Rumeysa Bilici Geçer, Derya Dursun

**Affiliations:** 1 Department of Orthodontics, Faculty of Dentistry, Istanbul Aydin University, İstanbul, TUR; 2 Department of Orthodontics, Faculty of Dentistry, Health Science University, İstanbul, TUR

**Keywords:** dental plaque, attitude, orthodontics, oral hygiene, periodontal diseases, white spot lesion

## Abstract

Aim: The aim of this study was to evaluate patients' attitudes and perspectives on the relationship between fixed orthodontic treatment and oral hygiene.

Material and methods: The study included 175 patients undergoing fixed orthodontic treatment. Data was collected through questionnaires that included patient demographics, oral hygiene behaviours, perspectives on white spot lesions (WSL) and periodontal problems, and attitudes towards orthodontic treatment. The chi-square test was used to analyse the relationship between categorical data. The significance level was set at p<0.05.

Results: The mean age of the patients (109 males, 66 females) was 17.8±6.4 years. Most of the patients who received orthodontic treatment brushed their teeth twice a day. Traditional and orthodontic toothbrushes were preferred over electric ones. The most common oral hygiene tools were toothbrushes and interdental brushes. A total of 151 patients (86.28%) indicated that their primary source of oral hygiene guidance was the orthodontist. A total of 113 patients (64.57%) had no knowledge of dental plaque, while 117 patients (66.85%) had no knowledge of WSL. While 80 patients (84%) with knowledge about periodontal problems believed that the primary cause of periodontal problems was inadequate oral hygiene, 43 patients (74.85%) with knowledge about WSL attributed the main cause of WSL to the use of braces. Negative attitudes towards orthodontic treatment and oral hygiene were more prevalent among adolescents (p*<*0.05), with no difference in gender or treatment duration. Patients who received information about WSL and periodontal problems exhibited a more positive attitude towards treatment and oral hygiene (p*<*0.05).

Conclusion: This study highlights the limited patient awareness of plaque and WSL during fixed orthodontic treatment. Physician guidance has a significant impact on patient attitudes, and specific educational programmes are required in orthodontic treatment.

## Introduction

Orthodontic treatment provides patients with functional occlusion, improved oral health, and an aesthetically pleasing smile [1]. The success of orthodontic treatment is contingent upon the expertise and abilities of the orthodontist, as well as the cooperation and compliance of the patient [2]. Patient cooperation is important throughout the long orthodontic treatment period and includes maintaining good oral hygiene [3,4]. Good oral hygiene is a key factor in maintaining orthodontic treatment and this can be controlled by the patient [5].

The most common oral diseases, dental caries, and periodontal disease are considered behavioural diseases because the adoption of healthy oral habits is crucial to controlling them [6]. It has been reported in the literature that orthodontic appliances make it difficult to maintain oral hygiene [7]. The irregular surfaces of brackets, bands, wires, ligatures, and other bonded appliances have been shown to increase plaque retention and limit the teeth's natural cleaning mechanisms, such as the action of the cheek muscles and the flow of saliva [8]. It is crucial to consider the increased plaque accumulation caused by orthodontic appliances, as microbial plaque is the primary etiological factor in dental caries and periodontal diseases, despite the different pathological mechanisms involved [9]. In the literature, the relationship between fixed orthodontic treatment and some adverse conditions such as white spot lesions (WSL) due to dental plaque, caries lesions showing cavitation with the progression of WSL, and periodontal problems such as gingivitis, bleeding, and alveolar bone loss has been reported [1,10]. Such complications have the potential to prolong or even terminate the orthodontic treatment process. The failure to prevent the adverse effects of poor oral hygiene during orthodontic treatment has the potential to become a significant public health concern. Therefore, patients undergoing orthodontic treatment should have good oral hygiene habits and be aware of the problems that can occur during orthodontic treatment.

It has been reported that problems caused by plaque accumulated during orthodontic treatment can be prevented through the implementation of effective oral hygiene education, patient awareness and motivation strategies [9]. It is crucial to consider the patient's perspective, attitude, and level of cooperation throughout the treatment process when evaluating the management system and the success of orthodontic treatment. Patients exhibit a range of knowledge levels and perspectives regarding oral hygiene habits and the potential risks associated with treatment. It has been reported that these differences may result from cultural changes, availability of oral health services, and different populations [11]. In order to develop effective oral hygiene programmes in orthodontic treatment, it is essential to evaluate both the oral hygiene habits and the perceptions of treatment risks and attitudes towards orthodontic treatment in the target group.

The aim of this study is to evaluate patients' attitudes and perspectives on the relationship between fixed orthodontic treatment and oral hygiene and their oral hygiene habits. The objectives of the study can be listed as follows: (i) To assess patients' knowledge of oral hygiene practices and potential oral health problems associated with fixed orthodontic treatment, (ii) To assess patients' attitudes towards oral hygiene and fixed orthodontic treatment, and (iii) To determine the factors (e.g. age, gender, treatment duration) that influence patients' attitudes and behaviours towards oral hygiene during fixed orthodontic treatment.

## Materials and methods

This was a cross-sectional study conducted with patients undergoing active fixed orthodontic treatment at the Orthodontics Department, Hamidiye Faculty of Dentistry, Health Science University in Istanbul, Türkiye. The survey was undertaken between January 2023 and June 2023. The study was approved by the Hamidiye Scientific Research Ethics Committee of Health Sciences University (approval number: 21/187). Informed consent was obtained from the patients and their parents and the study was conducted in accordance with the principles of the Declaration of Helsinki.

Study population and sampling

The sample size for the study was calculated as 175 using G-Power software version 3.1.9.2 (Heinrich-Heine- Universität, Düsseldorf, Germany) with an effect size of 0.5, 80% power, and a type I error rate of 5%. Data were collected and analyzed with a questionnaire applied to 175 patients receiving fixed orthodontic treatment. Inclusion criteria were patients with fixed orthodontic treatment for at least six months. Exclusion criteria were (i) patients whose orthodontic treatment was not continuing, (ii) patients with mental retardation or cognitive impairment, (iii) patients with craniofacial anomalies such as cleft lip and palate, and (iv) patients with orthodontic treatment for less than six months. 

Data collection

A web-based questionnaire was created using Google Forms (Google LLC, Mountain View, California, United States) and distributed via email to the patients in the patient files. Prior to the start of the survey, participants were provided with an informed consent statement, which explained the purpose of the study, the participation process, the privacy policy, and the voluntary nature of participation. This statement was prepared in accordance with ethical guidelines. At the end of the consent statement, participants were provided with a consent button to indicate their agreement to participate in the study. This button contained clear phrases such as 'I agree to participate'. By clicking on this button, participants gave their informed consent electronically.

The first part of the questionnaire included the patients' age, sex, and duration of treatment. The second part consisted of questions about the oral hygiene behaviour of the patients undergoing fixed orthodontic treatment. The third part included their knowledge about plaque, WSL, periodontal problems, and the instructions they received from their physicians about WSL and periodontal problems during the treatment process. The fourth part included patients' perspectives on WSL and periodontal problems in orthodontic treatment and attitudes towards the relationship between fixed orthodontic treatment and oral hygiene. The questions on patients' attitudes were adapted from a previous study [11] and were scored as 0 if the answer was negative and 1 if the answer was positive. Subjects were then divided into positive attitude groups (mean score 5-8) and negative attitude groups (mean score 0-4). Brochures on oral hygiene habits during orthodontic treatment were given to all patients who completed the questionnaire to increase awareness. The questionnaire form is provided in the Appendices.

Statistical analysis

Following the collection of data, the responses to the questionnaires were subjected to analysis. The statistical analysis was conducted using NCSS 2007 Statistical Software (NCSS, LLC., Kaysville, Utah, United States). The resulting values were calculated, and tables of results were prepared. The quantitative data were expressed in frequency and percentages. The chi-squared test was employed to detect differences in patient attitudes according to age group (under 18 years, adolescents and over 18 years, adults), gender and duration of treatment. The chi-square analysis was also used to determine the relationship between patients receiving instructions from their physicians and patient attitudes, with a significance level of p<0.05.

## Results

Data were collected from 175 patients (109 males and 66 females) undergoing fixed orthodontic treatment with their answers to the survey questions. The mean age of the patients was 17.8 ± 6.4 years. Of the total number of patients, 115 (65.71%) had received orthodontic treatment for a period of 6-18 months, while 60 (34.29%) had undergone treatment for a duration exceeding 18 months. 

When the oral hygiene habits of patients undergoing fixed orthodontic treatment were evaluated, 121 (69.14%) brushed twice a day, 31 (17.71%) brushed three times or more, and 23 (13.14%) brushed once a day. The most preferred toothbrushes were traditional/manual (50.28%) and orthodontic (40%), while electric toothbrushes (9.75%) were the least preferred. Table 1 illustrates the tools and methods employed by patients for oral hygiene during orthodontic treatment. Of the patients, 168 (96%) used toothbrush, 131 (74.85%) used interdental brush, 66 (37.71%) used fluoride toothpaste, 40 (22.85%) used fluoride mouthwash, 40 (22.85%) used dental floss, and 24 (13.71%) used mouth shower. The least preferred method was professional cleaning, preferred by only 12 patients (6.85%). A total of 151 patients (86.28%) indicated that they had acquired oral hygiene knowledge and habits from their orthodontist, 121 patients (69.14%) from their family, 63 patients (36%) from school, and 45 patients (25.71%) from social media. A total of 10 patients (5.71%) did not provide any information. 

**Table 1:** Responses regarding oral hygiene behaviour and source of information about oral hygiene in orthodontic treatment (more than one answer was possible)

Questions / Variables		Responses	n (%)
Oral Hygiene Behaviour	Frequency of tooth brushing	No brushing	0 (0.0)
		One time	23 (13.14)
		Two times	121 (69.14)
		Three and more	31 (17.71)
	Oral hygiene tools and methods in orthodontic treatment	Toothbrush	168 (96)
		İnterface brush	131 (74.85)
		Flouride toothpaste	66 (37.71)
		Dental floss	40 (22.85)
		Mouth shower	24 (13.71)
		Flouride mouthwash	40 (22.85)
		Professional cleaning	12 (6.85)
	Type of toothbrush	Manual toothbrush	88 (50.28)
		Orthodontic toothbrush	70 (40)
		Electric toothbrush	17 (9.75)
Source of information about oral hygiene	Where did you get information about oral hygiene habits?	Orthodontist	151 (86.28)
		Family	121 (69.14)
		School	63 (36)
		Social media	45 (25.71)
		No information	10 (5.71)

A total of 113 patients (64.57%) had no knowledge of dental plaque, while 117 patients (66.85%) had no knowledge of WSL. The perceptions of the patients regarding WSL and periodontal disorders were evaluated and are presented in Table 2. A total of 118 (67.42%) indicated that they had no knowledge about WSL that may occur during orthodontic treatment. Among the 57 patients (32.58%) who had knowledge about WSL, 43 (74.85%) attributed its primary cause to the use of braces, 36 (64%) to inadequate brushing and flossing, and 35 (62.28%) to difficulties in brushing during orthodontic treatment. A smaller proportion of patients (29.71%) attributed the cause to dental plaque. 

**Table 2:** Patients' perspectives about WSL and periodontal problems in orthodontic treatment (more than 1 answer was possible) WSL: white spot lesions

Questions	Responses	n (%)
Which of these can cause WSL?	Not brushing and flossing often enough (poor oral hygiene)	36 (64)
	Brushing difficulty in orthodontic treatment	35 (62.28)
	Dental plaque	17 (29.71)
	Braces	43 (74.85)
Which of these can cause periodontal problems?	Not brushing and flossing often enough (poor oral hygiene)	80 (84)
	Brushing difficulty in orthodontic treatment	53 (56)
	Dental plaque	24 (30.28)
	Braces	50 (53.14)

Eighty patients (45.71%) indicated that they had no knowledge about periodontal problems that may occur during orthodontic treatment. Among the 95 patients (54.29%) who had knowledge about periodontal problems, 80 (84%) attributed its primary cause to inadequate brushing and flossing, 53 (56%) to difficulties in brushing during orthodontic treatment, and 50 (53.14%) to the use of braces. A smaller proportion of patients (30.28%) attributed the cause to dental plaque. 

Attitudes towards the relationship between fixed orthodontic treatment and oral hygiene are given in Table 3. Among those exhibiting negative attitudes, the proportion of adolescents was higher (p<0.05). No gender or treatment duration differences were detected (Table 4).

**Table 3:** Patients' attitudes on the relationship between fixed orthodontic treatment and oral hygiene WSL: white spot lesions

Questions	Yes, n (%)	No, n (%)	
Fixed orthodontic appliance makes brushing more difficult	163 (93.15)		12 (6.85)
Are straight teeth easier to clean?	158 (90.29)		17 (9.71)
It is important to get oral hygiene advice and instructions from the orthodontist.	161 (92)		14 (8)
It is important to follow the oral hygiene advice and instructions given by the orthodontist.	126 (72)		49 (28)
It is important to brush more after wearing a fixed orthodontic appliance	140 (80)		35 (20)
Compliance with oral hygiene habits in orthodontic treatment prevents risks	124 (70.86)		51 (29.14)
Fixed orthodontic appliance initiate/cause periodontal problems?	84 (48)		91 (52)
Fixed orthodontic appliance initiate/cause WSL?	126 (82)		49 (28)

**Table 4:** Patients' attitudes according to gender, age group, and duration of treatment p<0.05 is indicated as statistically significance; Chi-square test were performed

Patients' attitudes	Sex, n (%)		Age, n (%)		Treatment duration, n (%)		Total, n (%)
	Male	Female	Adolescents	Adults	<18 month	>18 months	
Negative attitude Scores (0-4)	23 (13.14)	27 (15.42)	45 (25.71)	5 (2.85)	30 (17.14)	20 (11.42)	50 (28.57)
Positive attitude Scores (5-8)	43 (24.57)	82 (46.85)	61 (34.85)	64 (36.57)	91 (52)	34 (19.42)	125 (71.43)
P-value	0.15		0.000		0.09		-

A total of 114 (65.14%) patients indicated that they had not received instructions on the development and prevention of WSL. In contrast, 61 patients (34.86%) reported having received such instructions. Similarly, 117 patients (66.85%) stated that they had received instructions on the development and prevention of periodontal problems, while 58 patients (33.15%) indicated that they had not received such instructions. Patients who received information about WSL and periodontal problems exhibited a significantly more positive attitude towards fixed orthodontic treatment and oral hygiene (p<0.05) as shown in Table 5.

**Table 5:** Patient attitudes and information from physician p<0.05 is indicated as statistically significance; Chi-square test were performed

Questions	Responses	Positive attitude, n (%)	Negative attitude, n (%)	P-value
Have you received information about periodontal problems in orthodontic treatment?	Yes	112 (64)	5 (2.85)	0.000
	No	13 (7.42)	45 (25.71)	
Have you received information about the WSL in orthodontic treatment?	Yes	56 (32)	5 (2.85)	0.000
	No	69 (39.42)	45 (25.71)	

## Discussion

Good oral hygiene in patients undergoing orthodontic treatment requires adequate equipment, correct technique, regular brushing, and sufficient time to brush each tooth. When oral hygiene habits were evaluated in the study, most of the patients (69.14%) brushed their teeth twice a day. It has been reported in the literature that brushing at least twice a day is required for good oral hygiene during orthodontic treatment [12].

The most preferred oral care tools were toothbrush (96%), interface brush (74.85%) and fluoride toothpaste (37.71%). On the contrary, dental floss (22.85%), mouth shower (13.71%) and fluoride mouthwash (22.85%) were less preferred. Patients undergoing orthodontic treatment require the combined use of different oral hygiene tools [10]. It is important to encourage the use of additional hygiene tools such as mouth shower for effective cleaning, fluoride mouthwash to prevent enamel demineralization and dental floss specially made for orthodontic patients. Re et al. reported that in areas where plaque removal is difficult, the cleaning efficiency of mouth showers is higher compared to manual brushing [13].

The type of toothbrush (manual or electric) is one of the factors influencing brushing efficiency [14]. Today, conventional toothbrushes, electric toothbrushes, and ultrasonic toothbrushes of many different designs are used to effectively remove plaque [15,16]. In the study, traditional and orthodontic manual toothbrushes were more preferred, while electric toothbrushes were less preferred (9.75%). For economic reasons, the use of manual toothbrushes may be by far the most appropriate option for oral hygiene. Erbe et al. reported that electric toothbrushes with orthodontic heads increased internal motivation and plaque removal efficiency [8]. Conversely, some studies have reported that there is no difference between electric and manual toothbrushes in terms of plaque removal efficiency and reduction of gingivitis in adolescents undergoing fixed orthodontic treatment [17]. Regardless of the type of toothbrush used in orthodontic treatment, it is considered more important for professionals to focus on improving their patients' oral hygiene. In addition, professional recommendations can be made by considering the individual abilities and motivations of patients.

In the study, most of the patients (86.28%) stated that they learnt oral hygiene information and habits from their orthodontist, while a significant rate (25.71%) learnt from social media. Traditionally, the physician has been the main source of information about a patient’s diagnosis and treatment [18]. However, with the development of the internet and social media, the volume of data on the internet as a source of information may increase and change the doctor-patient relationship. Therefore, the accuracy of information on the internet and social media is important. Information on the internet and social media is not always regulated; this represents a challenge for physicians treating misinformed patients who believe what they read on the Internet and social media instead of their physician’s opinion [18]. Orthodontists can refer patients to the social media sites of orthodontic associations or the websites of orthodontic journals for reliable information. 

Remarkably, most of the patients (64.57%) in the study stated that they had no knowledge about dental plaque. Similarly, Alhajia et al. reported that most orthodontic patients do not know what plaque is and how it is caused [11]. The main cause of dental plaque accumulation is a lack of oral hygiene or inadequate or improper oral hygiene. Patients usually notice calculus and stains more easily than dental plaque. Dental plaque is more difficult to identify because it is tooth-coloured or colourless [11,19]. Patients may ignore plaque because it is less noticeable. Olivera et al. reported that regular visualization of plaque retention areas can improve patients' visual memory and brushing skills [19]. 

Most patients (66.85%) said they had no knowledge of WSL. When the perspectives of patients with knowledge of WSL were evaluated, most indicated that WSL developed because of braces. When the perspectives of patients with knowledge of periodontal problems were evaluated, most patients indicated that periodontal problems developed because of poor oral hygiene (Table 2). The education given by patients on the importance, aetiology, and prevention of WSL can prevent problems affecting aesthetics and dental health that may occur in orthodontic treatment. Studies have shown that patients with better knowledge of orthodontics have improved attitudes towards orthodontic treatment, thus increasing the likelihood of improved clinical outcomes [20,21].

WSL is less noticeable than periodontal problems such as gingival enlargement and bleeding. It is easy to miss early WSL unless archwires, ligatures, elastomeric chains, or other appliances are removed, and the teeth are free of plaque and debris and dried. The presence of gingival inflammation, which reduces the amount of visible enamel between the bracket and the gingival margin, can also make it difficult to visualize WSL during treatment. Therefore, when giving information about WSL, it may be more effective to give information through attractive visuals.

The study found that most adolescent patients had a negative attitude (p<0.05). This may be because children and adolescents pay less attention to information about oral hygiene and plaque. Many studies have reported that adults are more likely than children to comply with oral hygiene recommendations and warnings from physicians [22,23]. Visualizing areas of plaque and biofilm with chewable tablets or soluable plaque-disclosing agents can help educate and motivate patients, leading to improved awareness in children [19,24]. In children and adolescents, patient motivation for oral hygiene is important because if oral hygiene skills are learned and maintained during childhood, oral hygiene habits will be established that should last a lifetime [25]. 

In the current study, a significant proportion of the patient population, amounting to 114 individuals (65.14%), indicated that they had not received instructions on the development and prevention of WSL. However, patients may not remember receiving instructions at the beginning of treatment. Similarly, Maxfield et al. reported that a significant proportion of patients did not remember receiving instructions about the development of WSL [26]. Enamel decalcifications, or WSL, are the most frequent complication of fixed appliance therapy [26]. Richter et al. reported that an alarming 72.9% of patients developed at least one WSL during orthodontic treatment, of which 2.3% showed invitations [27]. Therefore, patients who notice WSL after the removal of brackets and bands after orthodontic treatment may be disappointed, which affects the success of the treatment.

The medical literature documents that physicians are responsible for informing the patient what is wrong, how it came about, how serious it is, and presenting the various treatment options [26]. In the literature, it was reported that six months after the consent form was obtained, patients remembered the reason, duration, and applications of the treatment at a high rate, but the rate of remembering the risks that may occur during treatment (caries, root resorption) and retention was low [28]. Instructions about things to consider during treatment and problems that may occur are usually explained to patients at the first treatment appointment. However, patients may ignore or forget this because they are more interested in their new device and aesthetics [29]. It is thought that providing information as an additional appointment or with regular reminders during treatment will be more effective in educating patients. 

The study found that patients who were informed about WSL and periodontal problems had a significantly more positive attitude towards fixed orthodontic treatment and oral hygiene (Table 5). This indicates that providing information can have a positive impact on patients' attitudes. Therefore, information provision strategies can be effective in promoting positive attitudes and increasing awareness in the community. 

In light of these findings, comprehensive patient education by orthodontists should be an integral part of treatment. By increasing patient awareness and understanding of the potential risks associated with fixed orthodontic appliances, particularly WSL and periodontal issues, we can foster more positive attitudes towards treatment and encourage better oral hygiene practices. Future research and clinical efforts should focus on developing and implementing tailored educational programmes that address these specific gaps in patient knowledge, ultimately leading to improved oral health outcomes and greater patient satisfaction. The time to act is now. By investing in patient education, we will not only improve the individual treatment experience but also contribute to the overall advancement of orthodontic care.

A major limitation of this study is the relatively small sample size, which was drawn from a single, homogeneous population. This limits the generalizability of our findings, as the results may not be applicable to broader, more diverse populations. In addition, the focus on a specific population may have overlooked important variables or differences that may be present in other demographic or geographic groups. To address these limitations, future research should aim to include a larger and more diverse sample, representing different socioeconomic backgrounds, cultural practices, and oral health behaviors. Expanding research in this way would provide a more comprehensive evaluation of oral health programmes, helping to determine their effectiveness in different populations and settings. This broader approach would also contribute to the development of more universally applicable oral health interventions.

## Conclusions

The majority of patients undergoing fixed orthodontic treatment are unaware of the presence of dental plaque and WSL. Educating patients about oral hygiene, risks, and benefits of orthodontic treatment is crucial for achieving positive outcomes and improving overall oral health. Physicians play a vital role in providing patients with the necessary information and guidance to instil good oral hygiene habits. Tailored health education programs targeted at different age groups can help shape positive attitudes towards treatment and enhance patient compliance. Emphasizing the importance of oral hygiene, providing personalized instructions, and ensuring regular follow-up appointments can lead to better treatment outcomes and fewer post-treatment complications. By prioritizing patient education and involvement, healthcare providers can contribute to the success of orthodontic treatments and promote overall oral health and well-being.

## Appendices

Survey questionnaire

**Table 6:** Questionnaire form

Demographic data of the participants
Q1 Gender
Male
Female
Q2 Age
Q3 Treatment duration
6-18 months
18 months

Oral hygiene behaviour of patients undergoing fixed orthodontic treatment
Q4 How often do you brush your teeth?
No brushing
One time
Two times
Three and more
Q5 Which oral hygiene tools and methods do you use in orthodontic treatment? (more than 1 answer was possible)
Toothbrush
İnterface brush
Flouride toothpaste
Dental floss
Mouth shower
Floruide mouthwash
Professional cleaning
Q6 Which type of toothbrush do you use?
Manual toothbrush
Orthodontic toothbrush
Electric toothbrush
Q7 Where did you get information about oral hygiene habits? (more than 1 answer was possible)
Orthodontist
Family
School
Social media
No information

Patients’ knowledge
Q8 Do you have knowledge about dental plaque? Yes/No
Q9 Do you have knowledge about WSL? Yes/No
Q10 Do you have knowledge about periodontal problems? Yes/No
Q11 Have you received information from your physician about the periodontal problems in orthodontic treatment? Yes/No
Q12 Have you received information from your physician about the WSL in orthodontic treatment? Yes/No

Patients' perspectives
Q13 Which of these can cause WSL? (more than 1 answer was possible)
Not brushing and flossing often enough (poor oral hygiene)
Brushing difficulty in orthodontic treatment
Dental plaque
Braces
Q14 Which of these can cause periodontal problems? (more than 1 answer was possible)
Not brushing and flossing often enough (poor oral hygiene)
Brushing difficulty in orthodontic treatment
Dental plaque
Braces

Patients' Attitude
Q15 Please tick yes/no to the following sentence/questions.
Fixed orthodontic appliance makes brushing more difficult. Yes/No
Are straight teeth easier to clean? Yes/No
It is important to get oral hygiene advice and instructions from the orthodontist. Yes / No
It is important to follow the oral hygiene advice and instructions given by the orthodontist. Yes / No
It is important to brush more after wearing a fixed orthodontic appliance. Yes / No
Compliance with oral hygiene habits in orthodontic treatment prevents risks. Yes / No
Fixed orthodontic appliance initiate/cause periodontal problems? Yes / No
Fixed orthodontic appliance initiate/cause WSL*? Yes / No
